# Comparative Study of Efficacy of Dopaminergic Neuron Differentiation between Embryonic Stem Cell and Protein-Based Induced Pluripotent Stem Cell

**DOI:** 10.1371/journal.pone.0085736

**Published:** 2014-01-22

**Authors:** Yoo-Wook Kwon, Yeon-Ju Chung, Joonoh Kim, Ho-Jae Lee, Jihwan Park, Tae-Young Roh, Hyun-Jai Cho, Chang-Hwan Yoon, Bon-Kwon Koo, Hyo-Soo Kim

**Affiliations:** 1 National Research Laboratory for Stem Cell Niche, Seoul National University Hospital, Seoul, Korea; 2 Innovative Research Institute for Cell Therapy, Seoul National University Hospital, Seoul, Korea; 3 Division of Molecular and Life Sciences,Pohang University of Science and Technology, Pohang, Korea; 4 Cardiovascular center, Seoul National University Bundang Hospital, Seoul National University, Seoul, Korea; 5 Department of Internal Medicine, Seoul National University, Seoul, Korea; 6 Molecular Medicine and Biopharmaceutical Sciences, Seoul National University, Seoul, Korea; University of Kansas Medical Center, United States of America

## Abstract

In patients with Parkinson's disease (PD), stem cells can serve as therapeutic agents to restore or regenerate injured nervous system. Here, we differentiated two types of stem cells; mouse embryonic stem cells (mESCs) and protein-based iPS cells (P-iPSCs) generated by non-viral methods, into midbrain dopaminergic (mDA) neurons, and then compared the efficiency of DA neuron differentiation from these two cell types. In the undifferentiated stage, P-iPSCs expressed pluripotency markers as ES cells did, indicating that protein-based reprogramming was stable and authentic. While both stem cell types were differentiated to the terminally-matured mDA neurons, P-iPSCs showed higher DA neuron-specific markers' expression than ES cells. To investigate the mechanism of the superior induction capacity of DA neurons observed in P-iPSCs compared to ES cells, we analyzed histone modifications by genome-wide ChIP sequencing analysis and their corresponding microarray results between two cell types. We found that Wnt signaling was up-regulated, while SFRP1, a counter-acting molecule of Wnt, was more suppressed in P-iPSCs than in mESCs. In PD rat model, transplantation of neural precursor cells derived from both cell types showed improved function. The present study demonstrates that P-iPSCs could be a suitable cell source to provide patient-specific therapy for PD without ethical problems or rejection issues.

## Introduction

Progressive degeneration of midbrain dopaminergic neurons (mDA) is one of major pathological causes in Parkinson's disease (PD). Since the clinical progression of PD cannot be effectively prevented, replacement of damaged cells by cell transplantation has become one of the promising therapeutic strategies. Since in the late 1980s, transplantation of human fetal ventral mesencephalic tissues into the striatum of PD patients has been adopted as a successful therapy for patients with advanced disease [1], [2]. However, this fetal brain tissue transplantation has serious hurdles such as ethical issue and the limited supply of fetal tissues. To circumvent these difficulties, several investigators utilized neurons with DA phenotype generated from embryonic stem cells (ESCs), the induced pluripotent stem cells (iPSCs), or neural stem cells (NSCs) as a practical and an effective alternative to the fetal brain tissues. Among these, DA neurons derived from ESCs were tested in PD animal models and proved to be effective in functional recovery. However, use of ESCs faces certain ethical and technical limitations because of their origin from human embryo [3], and possibility of immune incompatibility [4], [5]. iPSCs were able to generate DA neurons as well [6], although for iPSCs to be employed in clinical trials, there are still lot of tangled problems to solve such as developing methods to circumvent the use of pro-oncogene, *c-Myc*, and viral vector which serves as delivery system for iPS generation [7], [8].

Previously, we successfully produced iPSCs derived from mouse cardiac and skin fibroblasts by treating them with protein extracts derived from ESCs [9]. Unique strengths of our protein-based iPS cells (P-iPSCs) are its production without viral gene transfection or genetic manipulation and with easier and higher efficiency than previously reported [10], [11]. The protocol was simple, yet the P-iPSCs showed high development potential enough to produce whole fetal mice through germ-line transmission in the tetraploid complementation experiment. Unlike most of other iPS cell lines that incorporate lenti- or retro-viral systems, our P-iPSCs can be produced under the safer method by avoiding genetic manipulation. Therefore, we think our P-iPSCs as a good therapeutic source for PD.

Although several pluripotent stem cell-based therapeutics have actually entered clinical trials under United States Food and Drug Administration (FDA) jurisdiction and waiting to be approved [12], only 15% of patients showed improvement in previous clinical trials, while others have variable functional outcome due to the heterogeneity of cell quantity, quality, and engraftment rate as well as immune-suppression observed in patients [13], [14]. Therefore, it is necessary to engineer stem cells to obtain higher efficiency to differentiate into the desirable lineage, and to have ability to be engrafted into the tissues promptly without having any danger or ethical problems.

In this study, we compared ESCs and P-iPSCs in their ability to differentiate into DA neurons. Here, we demonstrate that the P-iPSCs show higher differentiation potential than ESCs and Wnt signals are closely associated with the efficiency of DA neuron differentiation. Furthermore, the induced DA neurons both from P-iPSCs and ESCs are effective in improving the symptoms of PD rats.

## Materials and Methods

### Maintaining Mouse ES Cells and Protein-iPS Cells

The cells were cultured as described previously [9]. In brief, STO feeder cells were used for following two mouse ES cell-lines. C57BL/6-background mouse ES cells (mESCs, accession #. SCRC-1002; ATCC), and 129/Ola strain-derived mouse ES cells (provided by Jeong Mook Lim, Seoul National University, Seoul, Korea). Protein-based-iPSCs were generated from mouse cardiac fibroblast (isolated from heart of C57BL/6 mice) and skin fibroblast (harvested from dermis of FVB mice) using ES cell-derived protein extracts (Protein-iPSCs). STO cells were cultured in Dulbecco's modified Eagle's medium (DMEM; GIBCO, Grand Island, NY) high glucose supplemented with 10% Fetal bovine Serum (FBS; GIBCO), and 100 U/ml penicillin Streptomycin (GIBCO). One day before subculturing mESCs or P-iPSCs, STO cells were treated with Mitomycin C (10 μg/ml medium, sigma-Aldrich, St Louis, MO) and seeded on a new 0.1% gelatin-coated dish. Propagating mESCs and P-iPSCs were cultured in DMEM (GIBCO) with 10% defined FBS (GIBCO), 2 mM L-glutamine (GIBCO), 1X NEAA (GIBCO), 1 mM 2-Mercaptoethanol (Sigma-Aldrich, St Louis, MO), and 100 units/ml Penicillin/100 μg/ml streptomycin (GIBCO) (ES media). In ES media, 2000 U/ml of ESGRO® LIF (leukemia inhibitory factor, Chemicon) was added to maintain pluripotency. mESCs and P-iPSCs were dissociated with 0.05% trypsin (GIBCO) and passaged on STO every 2∼3 days.

### In Vitro Differentiation of mESCs and P-iPSCs

Cells were differentiated into dopaminergic neurons using previously reported protocols [15]–[17]. Briefly, cells were trypsinized into single cells for 3∼5 minutes in room temperature (RT) and re-suspended with ES media in the absence of LIF on culture dish for 20 minutes to eliminate feeder cells. Only suspended cells were recollected and cell number was counted. To trigger differentiation, we used the formation of embryoid body (EB) through hanging drop method [18], [19]. About 350 cells were contained in each 20 μl of drop on non-adherent culture dishes. Cell-containing droplets were maintained in humidified 37°C, 10% CO_2_ incubator for 3 days. On the 4th day, the expanded cell drops were collected and attached on 1.5% gelatin-coated dishes for 24 hrs. For the selection of cells positive for nestin, which is a marker of neuronal precursor cells, cells were incubated in ITSFn medium (DMEM/F-12 media containing, Insulin (5 μg/ml), Apotranferrin (50 μg/ml), Sodium Selenite (30 nM), Fibronectin (250 ng/ml, all from Sigma), 100 U/ml penicillin, 100 μg/ml streptomycin(GIBCO)) [20]. Cells were cultured in this media for 7∼9 days which the media was replaced every 1∼2 days. Next, cells were detached from cell culture plates and neural precursor cells were plated onto dish coated with poly-L-ornnithine (15 μg/ml, Sigma-Aldrich)/Fibronectin (1 μg/ml, Sigma-Aldrich) at a density of 75,000 cells per cm^2^ [21]. After 24 hours, neural expansion was triggered by adding progesterone (20 nM), putrescine (100 nM), laminin (1 μg/ml) (all from Sigma-Aldrich), basic fibroblast growth factor (bFGF) (10 ng/ml, Invitrogen, Carlsbad, CA), Sonic hedgehog (500 ng/ml, R&D systems), basic fibroblast growth factor 8b (FGF 8) (100 ng/ml, R&D systems, Minneapolis, MN), 100 U/ml penicillin, and 100 μg/ml streptomycin (GIBCO) in ITSFn media (N3 media) for 4 days. Induction of terminal differentiation into dopaminergic neurons from expanded-neural precursor cells were performed by culturing precursor cells in neuronal-expansion media (N3 media containing 200 μM ascorbic acid instead of bFGF) for 8∼10 days.

### Measurement of migration distance from EBs

Undifferentiated mESCs and P-iPSCs were trypsinized and pre-incubated on non-adherent culture dishes, resulting in the formation of EBs as described above. After 3 days culturing in 37°C, 5% CO_2_ incubator, EBs were attached on 1.5% gelatin-coated culture dish for 24 hours. A distance from the margin of proliferating cells to edge of EBs was measured to compare cell proliferation rate respectively.

### RNA preparation and Real-Time RT-PCR Analysis

Total RNA from plated cells at each stage was purified using PureLink RNA extraction Kit (Invitrogen, Grand Island, NY) and 500 ng of RNA was transcribed into cDNA by using cDNA synthesis kit (Takara, Shiga, Japan). Conventional RT-PCR was carried out with Emerald Green PCR Master mix (Takara) on a S1000 Thermal cycler (GenDepot). Quantitative real-time RT-PCR (qRT-PCR) was done with SYBR Green master mix (Roche, Hague RD, IN) using Applied Biosystems 7500 Fast Real-Time PCR system. See Table S1 for the list of primers used to analyze target genes.

### Flow cytometry

Final stage of mDA differentiated cells was harvested and washed twice with cold PBS. Then, cells were fixed with 2% paraformaldehyde (WAKO chemical, Japan) at 4°C for 1hr then washed with FACS buffer (2.5% FBS, Gibco, in PBS). Finally, cells were incubated with the TH (Sigma-Aldrich) antibody for analysis. An IgG isotype antibody was used to define the percentage of positive cells. Analyses were performed using FACS CaliburTM (Becton Dickinson) and FlowJo software (FlowJo).

### Analysis of microarray and Genome-wide ChIP-Sequencing

Genes associated with dopaminergic neuron differentiation were selected using gene ontology terms from http://www.geneontology.org. Total of 185 genes, which are known to be related with dopaminergic neuron differentiation, were examined. The heat map was drawn by hierarchical clustering to compare the expression levels and degree of histone modifications between mESCs and P-iPSCs. The degree of H3K4me3 and H3K27me3 was calculated by normalizing the sum of ChIP-Seq tag reads from 1 kb upstream transcription start site to transcription end site by total sequencing tag reads.

### Methylation analysis via bisulfite sequencing

1 ug of genomic DNA was isolated from stage 3 cells of mESCs and P-iPSCs. Bisulfite DNA conversion was performed with EpiTect® Bisulfite kit (Qiagen, Valencia, CA) according to manufacturer's instruction. Converted DNA was used to amplify SFRP1 promoter region with specific primers according to reference [22]; left primer: GATTTGGGTTTAGTTTTAGTA, right primer: RATCRATAAAATCCTCCRCT in condition of 36 cycles of 94°C for 30 seconds, 56°C for 30 seconds, and 72°C for 60 seconds. Amplified DNA was ligated with pGEM®-T Easy Vector Systems (Promega, Madison, SI).

### Surgical procedure and Apomorphine-induced behavioral Analysis

All animal experiments were performed after receiving approval from the Institutional Animal Care and Use Committee (IACUC) of Clinical Research Institute in Seoul National University Hospital, Korea. Rat model of Parkinson's disease was generated by the previous procedure [23], [24]. Briefly, six weeks old adult female Sprague-Dawley rats (weighing 180–200 g) were pre-anesthetized, placed in a Kopf stereotaxic frame (Kopf Instruments, Tujunga, CA) and received unilateral stereotaxic injection of the 5 μl of 6–hydroxydopamine (6-OHDA, 8 μg/μl, Sigma-Aldrich) into the forebrain bundle (from the inter-aural: AP +0.42, ML +0.2, DV −2.80). After 3 weeks, animals were selected by rotational behavior in response to apomorphine (0.5 mg/kg, Sigma-Aldrich). Animals were placed (randomized) into automated rotormeter bowls, and monitored for 30 minutes to check rotational movements. Only the animals showing more than 6 ipsi-lateral rotations per minutes were regarded as stable model for cell transplantation.

### Cell transplantation, Histological procedure, immunofluorescence analysis

First, cells were tagged with fluorescent nanoparticle (NEO-STEM^TM^) by endocytosis process for 24 hours in prior to transplantation. Differentiated neural precursor cells were trypsinized at stage 3, day 7 and transplanted into the contralateral striatum of 6-OHDA-lesioned rats (from the bregma: AP +0.07, ML +0.30, DV −0.64). Each animal received 4 μL of 10^5^/μL cells diluted with 0.2% ascorbic acid in saline or 4 μL of PBS. Cells were injected by using 22-gauge Hamilton micro-syringe, at a rate of 1 μL/min and for 5 minutes to allow cells to settle down into tissue before needle was removed. Animals received vehicle (PBS) injection (n = 5), mESCs (n = 7) and P-iPSCs (n = 7). Efficacy of cell therapy was assessed as changes in apomorphine-induced behavior at 2, 4, 6 and 8 weeks after cell injection.

Rats were pre-anesthetized and sacrificed with intracardial perfusion fixation and brain was harvested. Sample was fixed in 4% formaldehyde at room temperature for 2 days and tissue was frozen for cryo-sectioning. Serials of coronal 40 μm-cryosections were kept in freezing medium until usage.

For immuno-fluorescent staining *in vitro*, cells in adequate stage of differentiation was prepared on μ-Dish *^35mm, high^* (ibidi, Germany), rinsed with PBS twice and fixed with 4% paraformaldehyde. For tissues, free-floating section staining was performed. Adequate sections of tissue were selected according to the atlas of Paxinos and Watson. After blocking for 1 hour, primary antibodies were added and incubated at 4°C for overnight. The following primary antibodies used in immuno-fluorescent staining: mouse anti-Tuj1 (Covance; 1∶500), mouse anti-Nestin (Chemicon; 1∶100), rabbit anti-Nurr1 (Santa Cruz; 1∶100), rabbit anti-Oct3/4 (Santa Cruz; 1∶50), rabbit anti-Pitx3 (Invitrogen; 1∶200), mouse anti-SSEA-1 (Santa Cruz; 1∶50), sheep anti-TH (Abcam, Cambridge, UK; 1∶2,000), goat anti-VMAT2 (Santa Cruz; 1;50), and goat anti-Wnt5a (Santa Cruz; 1∶50). Cells/tissues were incubated at room temperature for 1 hour with appropriate Alexa Fluor fluorescent-labeled secondary antibodies and washed with PBS. The 4, 6′-diaminobenzedine (Sigma-Aldrich; 1∶10,000) or sytox blue was used for counter staining, and cells/tissues were placed on Carl Zeiss LSM 710 to obtain confocal images.

### Statistical Analysis

Data are presented as mean ± standard error of the mean (SEM). Statistical analysis was performed by Student *t*-test or ANOVA as appropriate. SPSS version 18.0 was used for analysis. A probability value of <0.05 was considered statistically significant.

## Results

### P-iPSCs Possess More Potential to Differentiate Into Neuron Progenitor Cells Compared To Mouse ESCs

**Figure 1:**
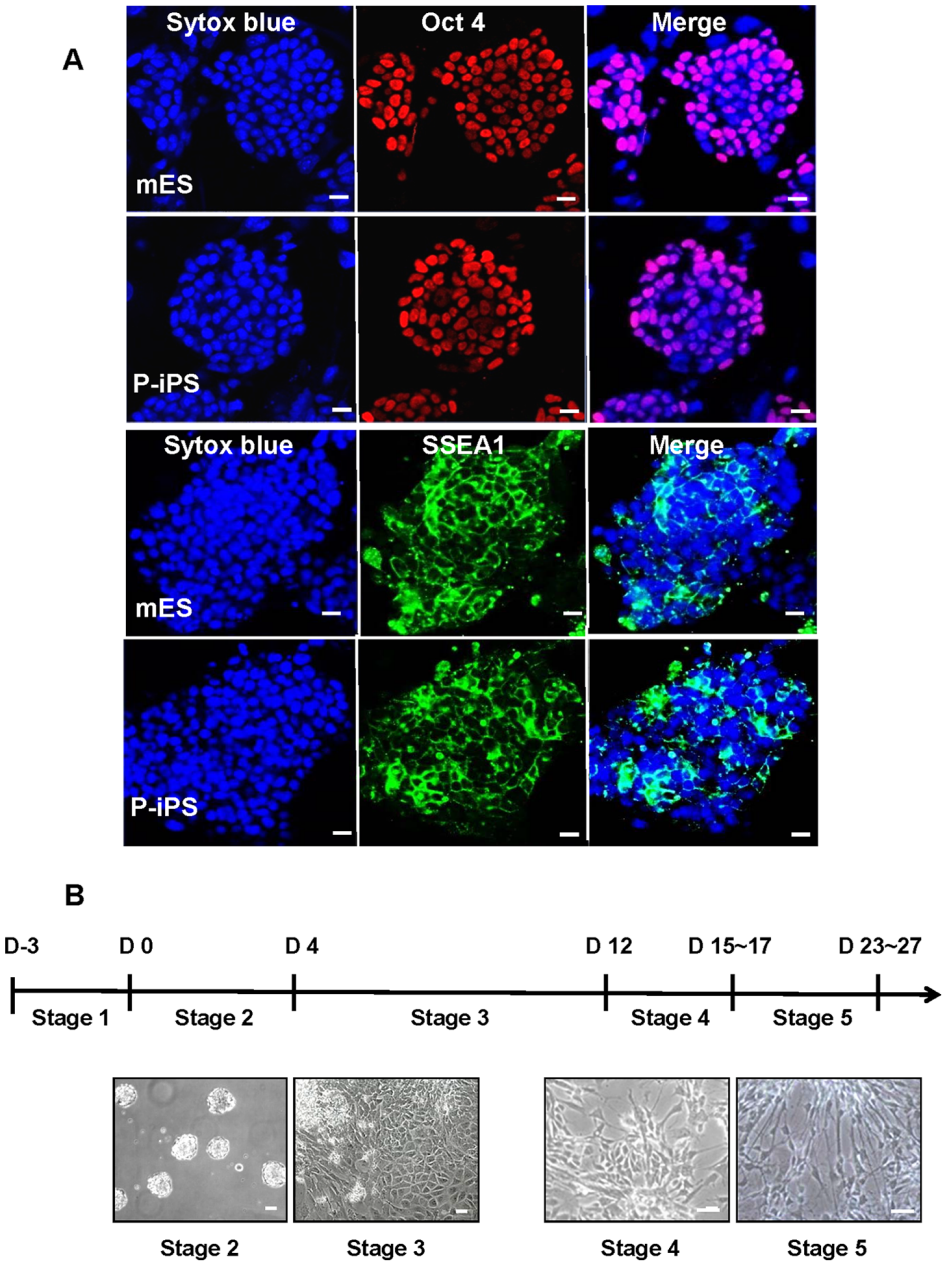
In vitro characterization of pluripotency of mESCs and P-iPSCs and schematic overview of experimental design for mDA neuronal differentiation. (A) Confocal images of mESCs and P-iPSCs to show pluripotency markers, Oct-4 and SSEA1 within cell colonies. Nuclei were stained with Sytox blue. Oct-4, and SSEA1 were expressed in nucleus or membrane respectively. Scale bars  = 20 μm. (B) DA neuronal differentiation is composed of five stages. Each stage showed distinct morphological changes in stem cells. The aggregated form of EBs was attached on gelatin-coated dish for 7 days. During this step, cells were transformed into tightly-packed epithelial morphology in ITSFn media. Propagation of neuronal precursor cells begins when cultured in N3 media for 3 to 5 days. Lastly, terminal differentiation into mDA neuron begins after culturing them in N3 media for 8 to 10 days in the absence of bFGF and addition of ascorbic acid in N3 media for 8∼10 days.

**Figure 2:**
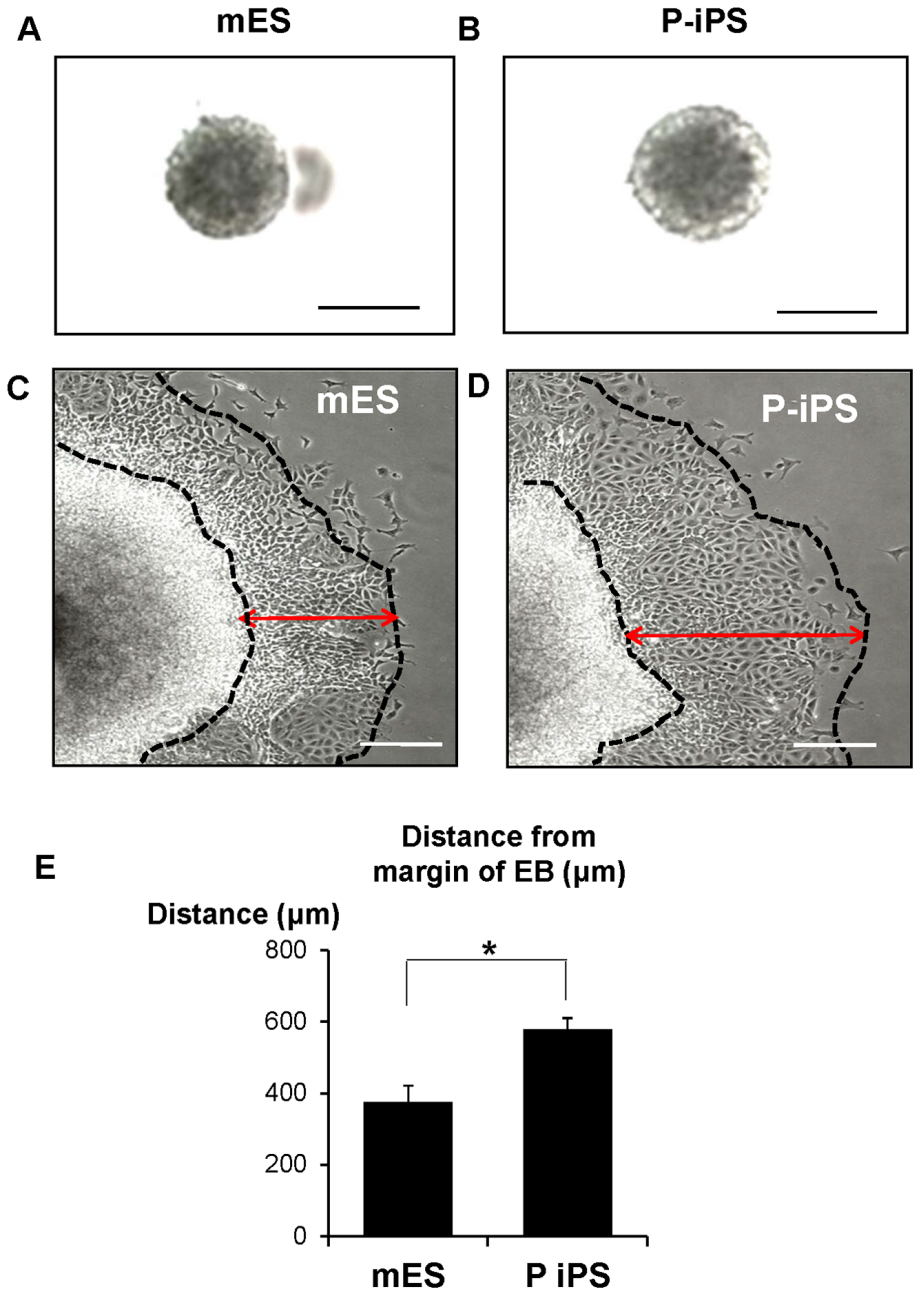
P-iPSCs-derived EBs have more potent migration capacity compared mESCs-derived EBs. (A, B) Microscopic image of mESCs and P-iPSCs in hanging drop culture for 4 days. Both mESCs and P-iPSCs formed similar spherical-shaped mass. (C–E) Sprouting cells from EBs were observed and the morphologically appearing largest migration distance from the outer region of aggregated EB to farthest region within the migration zone (indicated by black dashed-line and double-ended red arrow) was measured after 24 hours post-attachment. Data are presented as mean ± SEM. The symbol * denotes high statistical significance (P<0.05), mES vs P-iPS, All values are representative of three independent experiments, Scale bars  = 20 μm.

We first investigated the expression level of stemness markers, Oct4 and SSEA1, in mESCs and P-iPSCs to examine their pluripotency (Figure 1A). The expression of Oct4 and SSEA1 were evenly distributed across mESCs and P-iPSCs, confirming that both cells maintained homogeneous pluripotent states in prior to differentiation. Next, we induced differentiation of mESCs and P-iPSCs into DA neurons *in vitro* as described in method section. Each steps of differentiation were performed as described in a previous report [3] (Figure 1B). Undifferentiated cells (stage 1) were trypsinized and made into embryoid bodies (EBs) to eliminate self-renewal factors and to mimic embryogenesis *in vivo* [25] (stage 2). In order to control the size and shape of EBs, we used hanging drop method and were able to obtain the regularly-shaped EBs after 3 days (Figure 2A, B). Twenty-four hours after attaching EBs on gelatin-coated dish, cells migrated out from the attached EBs (Figure 2C, D). We previously reported that hypoxic priming accelerates vascular-lineage differentiation of mESCs and migration rate from EB aggregate center by hypoxia correlates to differentiation potential [26]. So, we measured the migrating distance from edge of EBs after 24 hours post-attachment. We observed a greater migration distance in P-iPSCs as compared to mESCs (mESCs; 375.9±45.0 μm, P-iPSCs; 578.3±32.1 μm, n = 3; (P<0.05)) (Figure 2E). Here, we expected that P-iPSCs might have a higher differentiation potential than ESCs. Next, we checked whether cultured EBs are able to differentiate into neural cells. To do so, both EBs at stage 3 of differentiation were plated in ITSFn media for seven days in order to select neural precursor cells [27] (stage 3). Using immuno-fluorescence staining, we observed that stage 3 cells derived from P-iPS were more nestin-positive compared to mESCs (Figure S1). Together, these results suggest that P-iPSCs intrinsically have a higher neuronal differentiation potential to differentiate into neural cells compared to ESCs.

### Development Markers of Dopaminergic Neurons Are More Highly Expressed in P-iPSCs-Derived Dopaminergic Neuron Cells Compared to ESCs

Next, neural precursor cells were expanded via culturing on dish coated with poly-L-ornithine and fibronectin in N3 media for 3∼5 days (stage 4) and dopaminergic neuron differentiation was induced by withdrawal of bFGF and the addition of ascorbic acid in N3 media (stage 5).

**Figure 3:**
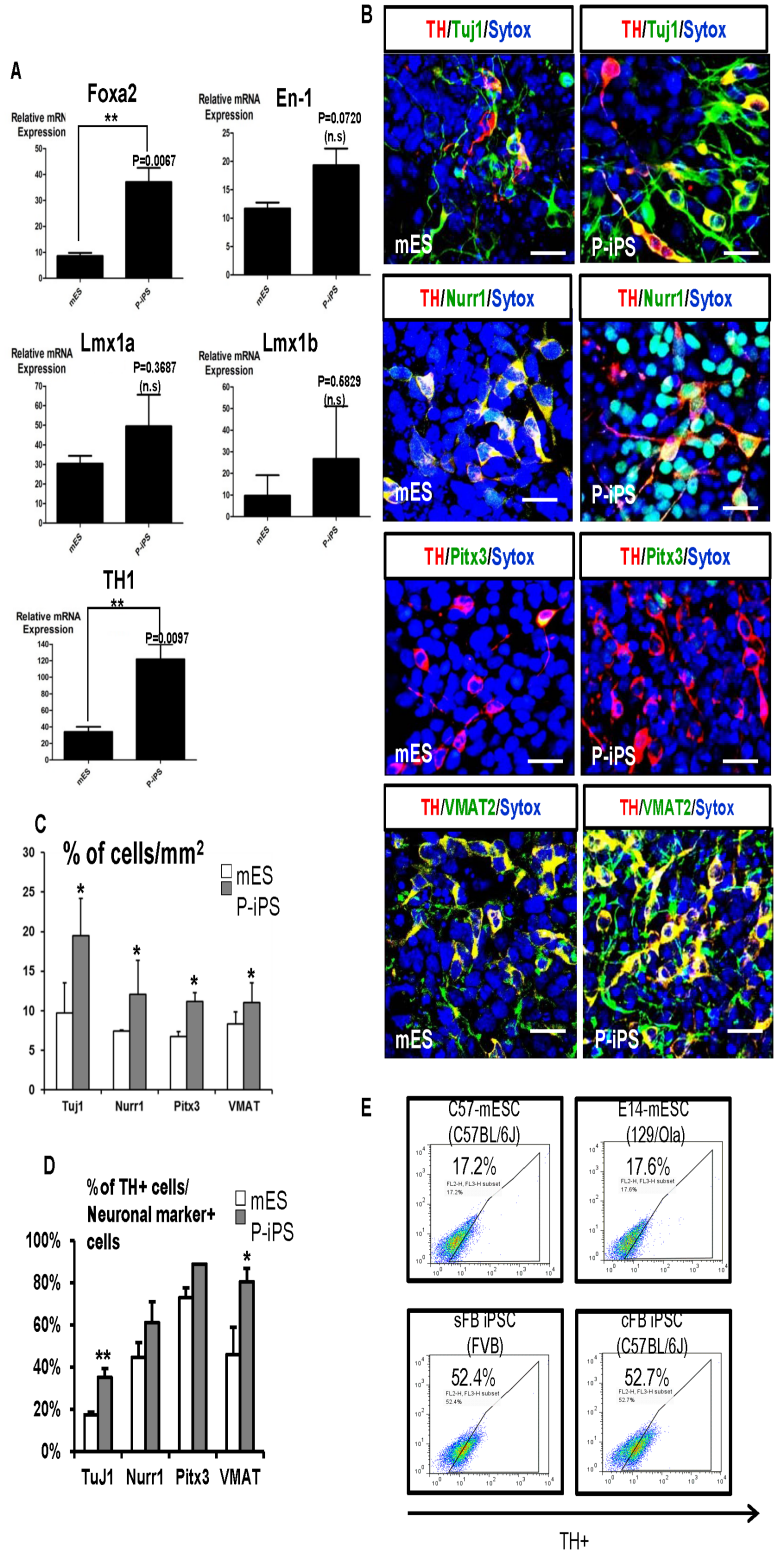
Comparison expression analysis of mouse DA neuronal specific markers between mESCs and P-iPSCs during neuronal differentiation. (A) Gene expression of previously reported mDA neuronal specific markers was confirmed by quantitative RT-PCR during neuronal differentiation. Following mRNA expression represents relative gene expression at stage 5 compared to stage 1. Most of gene expression of markers was relatively stronger in P-iPSCs than mESCs. These experiments were repeated three times. (B) Representative immunofluorescence data of mESCs and P-iPSCs at stage 5. Stronger TH-positive cell signals and more numbers of double-positive cells (TH/Tuj1 or/Nurr1 or/Pitx3 or/VMAT2) were observed in P-iPSCs than mESCs. Scale bars  = 20 μm. (C) Total cell numbers of mDA specific marker positive were counted in randomized fields (n = 10) out of 5,000cells, revealing that more abundant number of cells was counted in P-iPSCs. Data are presented as mean ± SEM (* P<0.05). All values are representative of 3 independent experiments. (D) The percentage of TH-positive cell number divided by neuronal marker-positive cell numbers was shown in bar graph. The bar graph shows the yield of terminal differentiation marker, TH-positive neurons from neural precursor cells. Data are presented as mean ± SEM (* P<0.05; ** P<0.01). All values are representative of three independent experiments. (E) Quantification of TH-positive cells between two different types of mESC and P-iPSC by FACS. C57-mESC is derived from C57BL/6 mouse strain. E14-mESC is derived from 129/Ola mice. Skin fibroblast (sFB)-derived P-iPSC is primarily cultured and generated from dermis of FVB mice. Cardiac fibroblast (cFB)-derived P-iPSC is originally obtained from C57BL/6 mice heart. Each group of cells at stage 5 mDA differentiation was harvested and expression of TH was analyzed in a quantitative manner.

To assess successful differentiation of mESCs and P-iPSCs into mDA neurons, we analyzed the expression of specific development markers of mDA neuron by quantitative RT-PCR [16] using RNA samples harvested at stage 1, and 5 from mESCs and P-iPSCs (Figure 3A). Following markers of mDA neurons were used, according to the previous reports which are known to be involved in the regulation of survival and maintenance of mDA neuron in developmental stages – *Foxa2*, *Lmx1a*, *Lmx1b and En1* [28,29.30] The gene expression of *Foxa2*, which regulates dopaminergic neuron generation and differentiation, was much higher in P-iPSCs than mESCs. Interestingly, P-iPSCs showed not too much but higher expression of both *Lmx1a* and *Lmx1b* genes compared to mESCs. Midbrain-hindbrain gene, *En1*, was expressed higher in P-iPSCs than mESCs throughout differentiation. Next, we quantified expression level of Tyrosin hydroxylase (TH), which is a golden standard method to determine whether mDA neuron differentiation was successful. As expected, we confirmed that *TH* expression was significantly higher in P-iPSCs than mESCs at stage 5. Next, we performed immunofluorescence analysis with various antibodies against Nurr1, Pitx3 (expressed in dopamine neurons), and vesicular monoamine transporter2 (VMAT2) to explore differentiation ability of mESCs and P-iPSCs at protein expression (Figure 3B). The expression of all these markers was merged with TH expression in cells between 7 to 11 days after stage 5. We observed double-positive cells for TH and Tuj1 which detects β-III tubulin at higher frequency in P-iPSCs than mESCs (Figure 3C). Similar results were obtained with double-labeled TH-positive neurons after staining with other regional specific markers including Nurr1, Pitx3 and VMAT2. Majority of TH-positive cells exhibited a similar morphology of midbrain dopamine neuron, indicating their regional specification *in vitro*. We quantified the number of cells positive for mDA neuron marker in. As predicted, P-iPSC groups showed higher number of cells positive for regional specific marker than mESC groups (Figure 3D). Strikingly, we observed that the expression pattern of tyrosine hydroxylase (TH), which is a golden standard of mDA neuron differentiation marker, was much higher in both iPS cell-lines compared to two different ES cell-lines at stage 5 mDA differentiation (Figure 3E). Therefore, we believe that our P-iPS cell-lines possess intrinsic cellular characteristics, leading to higher capacity to differentiate into mDA neurons compared to traditional mESCs. Taken together, these *in vitro* data suggest that P-iPSCs could differentiate into mDA neurons in higher efficiency than mESCs depending on expression of genes related in mDA neuron development.

### Activation of the Wnt signaling and Inhibition of its Counter-actor SFRP Lead to Higher Ability to Differentiate into mDA neurons in P-iPSCs than in mESCs

**Figure 4:**
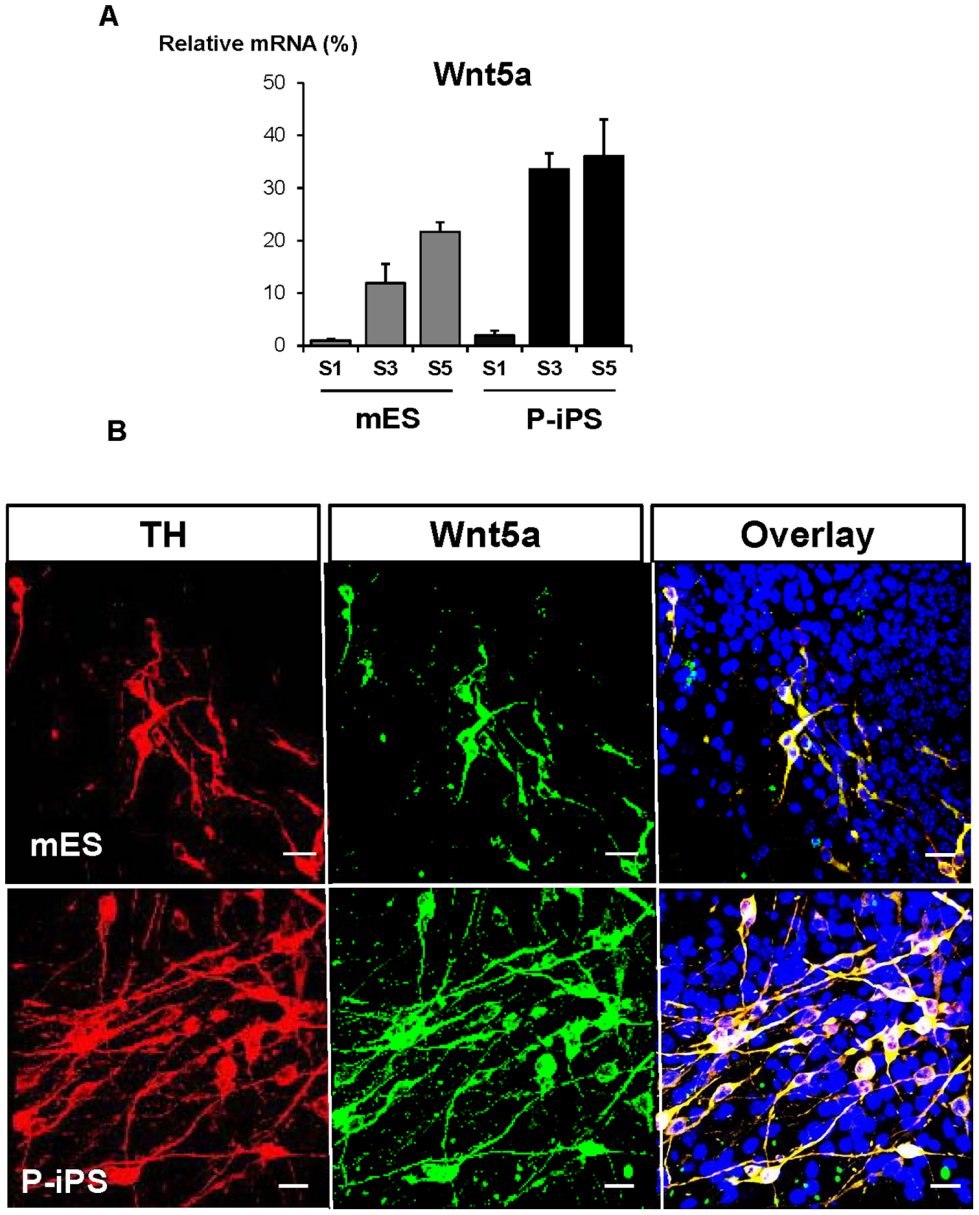
Comparison of Wnt5a expression between mESCs and P-iPSCs during neuronal differentiation. (A) Gene expression of Wnt5a at stage 1,3 and 5 during differentiation between mES and P-iPS cells was confirmed by real-time PCR. While Wnt5a mRNA levels in mESCs were gradually increased, the signals in P-iPSCs were rapidly increased from S3, resulting in the higher Wnt5a expression in S5 of P-iPSCs than mESCs. (B) Immunofluorescent staining of TH and Wnt5a between mES and P-iPS at stage 5. Immunofluorescence data reveal more Wnt5a–positive cells leads to have higher TH–positive protein expressions in P-iPSCs than in mESCs. Scale bars  = 20 μm.

In order to investigate the mechanism of higher efficiency in differentiation to DA neuron observed in P-iPSCs, we compared gene expression pattern between mESCs and P-iPSCs using microarray analysis and the ChIP-Seq profiles for histone modifications. We analyzed differential expression levels and histone modification patterns of 185 genes which are involved in dopaminergic neuron differentiation selected from the Gene Ontology database (Figure S2A). In our analysis, we targeted Wnt signaling related components since it is well-known to be involved in development of the nervous system by both canonical and non-canonical signaling pathways. Among Wnt families, we focused on Wnt5a because it was dramatically increased in P-iPSCs than mESCs. Moreover, H3K4 trimethylation in Wnt5a promoter, which is associated with transcription activation, was also highly enriched in P-iPSCs (Figure S2B). To quantify and examine gene expression pattern of Wnt5a, we performed real-time PCR using these cells at stage 1, 3, and 5. As expected, Wnt5a was gradually increased during differentiation, with a higher expression observed in P-iPSCs than ESCs at stage 3 and stage 5 (Figure 4A). Since Wnt5a is known to be a soluble factor to regulate ventral midbrain morphogenesis and to promote the differentiation of Nurr1-positive cells into TH-positive cells [28]–[30], we investigated whether there is a correlation between Wnt5a and TH expression in both cells. Interestingly, Wnt5a and TH were highly co-localized and expressed in P-iPSCs more than in mESCs (900±166.7/mm^2^ vs 392±66.7/mm^2^ in three random fields), suggesting that Wnt5a is an important upstream regulator mediating differentiation to TH-positive neurons (Figure 4B & Figure S3A). These results illustrate that superior efficiency in differentiation to mDA neuron observed in P-iPSCs may be related with the higher expression of Wnt5a. In genome-wide ChIP-Seq analysis of all genes involved in Wnt signaling pathway, there was no notable differences in H3K4 trimethylation level between mESCs and P-iPSCs even though some Wnt family genes showed elevated H3K4 trimethylation level. However, the H3K27 trimethylation enrichment was higher in mESCs than P-iPSCs at gene body regions and upstream of transcription start sites (Figure S2C). The different pattern of histone repression mark in both cells at promoter sites suggested that differentially down-regulated genes might be present during mDA neuron differentiation.

**Figure 5:**
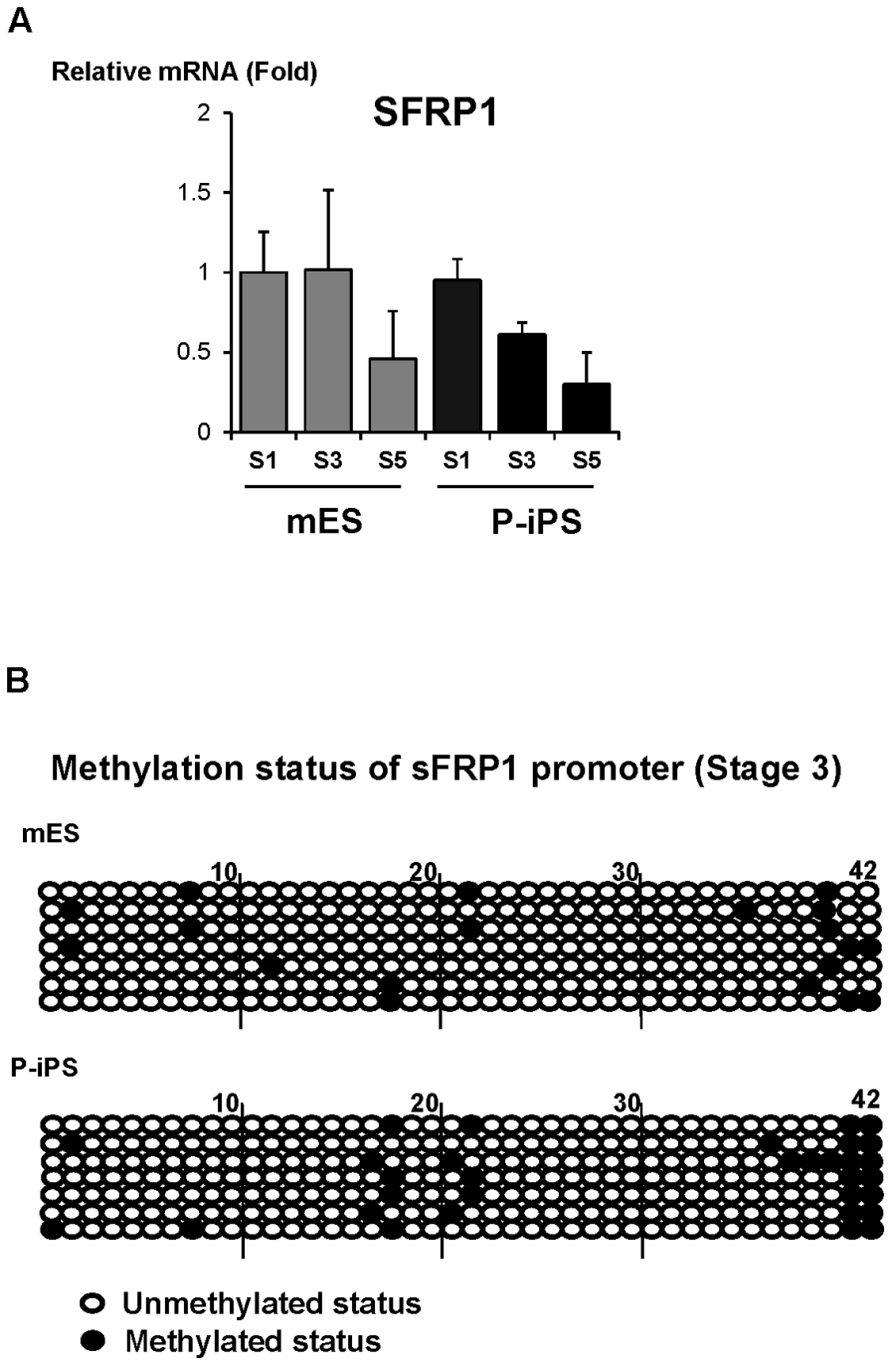
Expression level of SFRP and methylation status of its promoter during differentiation stages of mESCs and P-iPSCs. (A) mRNA expression of SFRP1 at each differentiation stages was analyzed in both mES and P-iPS cells during differentiation. The SFRP1 gene expression in three representative stages of cells was down-regulated as cells become terminally differentiated. Lower expression levels of SFRP1 gene was maintained from S3 in P-iPSCs than mESCs. Each gene expression was normalized to that of GAPDH expressions and presented relative to the respective value of the S1 mES levels. (n = 3) (B) Bisulfite sequencing of sFRP1 promoter of mES and P-iPS cells during stage 3 differentiation. Out of 42 CpGs, hypermethylation at 17^th^, 18^th^, 21^st^, and 22^nd^CpGs of SFRP1 promoter was observed between mES and P-iPSCs.

We next screened for genes that are related to Wnt signaling with decreased expression level in P-iPSCs compared to ESCs. To our surprise, mRNA expression of the secreted Frizzled-related proteins 1 (SFRP1) was notably higher in ESCs than in P-iPSCs at stage 1 and evidently much higher at stage 3 and 5 (Figure S3B). SFRP1 is one of factors to inhibit both canonical and non-canonical Wnt signaling by reversibly preventing Wnt from interacting with membrane-bound receptors [31], [32]. There was a strong expression of SFRP1 at stage 1 and stage 3 during differentiation in mESCs. Interestingly, we observed a more dramatic down-regulation of SFRP1 expression in P-iPSCs-derived neural precursor cells, even though that SFRP1 expression was gradually decreased in both types of cells during differentiation (Figure 5A & Figure S3B). Since DNA methylation of promoter generally down-regulates the gene expression, we investigated the DNA methylation status of the SFRP promoter in the mESCs and P-iPSCs. By performing bisulfite sequencing analysis, we observed a slight increase in methylation of SFRP promoter and gene body in P-iPSCs relative to mESCs (Figure 5B). These data suggests that methylation sensitivity and histone modification status of SFRP gene regulatory region control the expression of SFRP and that the decreased level of SFRP might augment the signal transduction of Wnt5a and differentiation to mDA neurons in P-iPSCs.

### Successful Engraftment of P-iPSCs-derived Neural Precursor cells Relieved Symptom in Parkinsonian Rats

**Figure 6:**
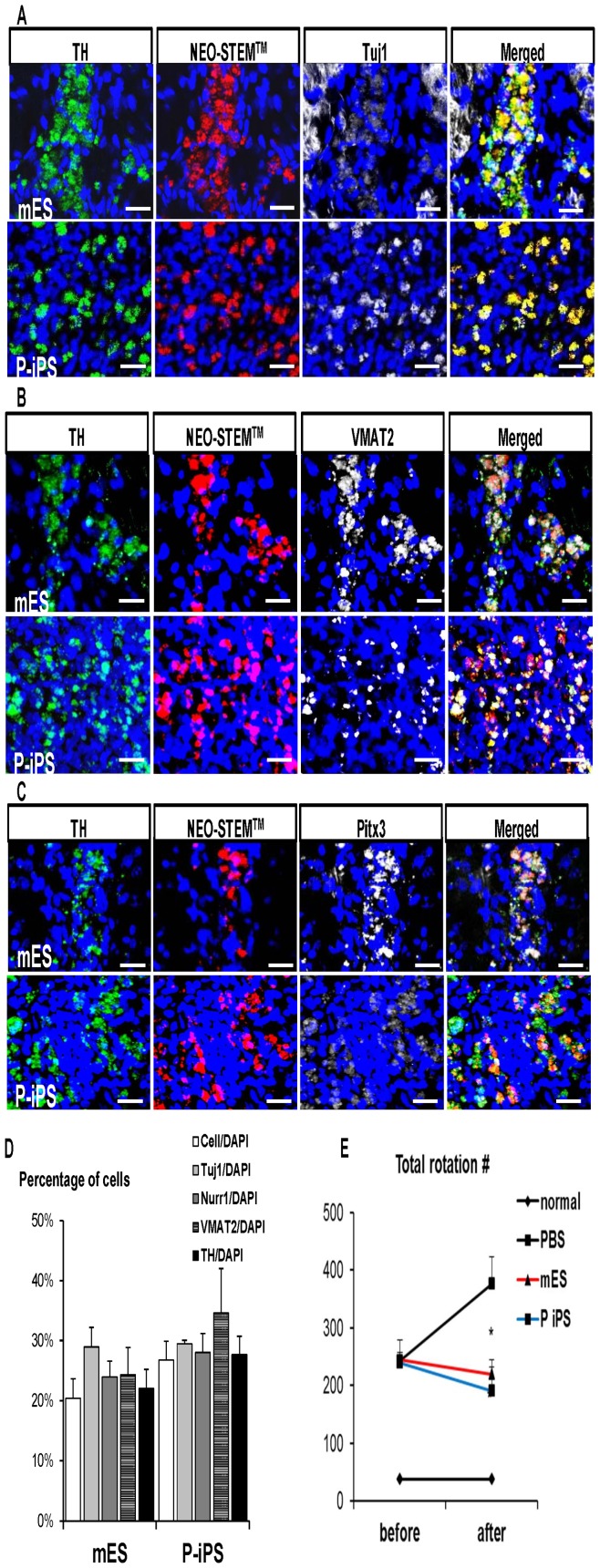
Transplantation of neuronal precursor cells of mESCs and P-iPSCs In vivo Parkison's disease model of rats. (A) Nano particle-labeled injected cells are shown as red which were observed in the graft region. Majority of TH-positive cells (green) were also Tuj1 (white),(B) VMAT2 (white) and (C) Pitx3-positive cells (white). Scale bars  = 20 μm. (D) Quantitative analysis of cells counted against nucleus marker, DAPI in three random fields revealed that higher number of cells existed with neuronal-positive signals in P-iPSCs when compared to mESCs injected groups. (E) Total apomorphine-induced rotation numbers (0.5 mg/kg) were counted at two weeks after cell injection. mESC (n = 7) and P-iPSC injected groups (n = 7) showed the improved symptom compared to PBS injected groups (n = 4), while average rotation score of P-iPSC group was slightly decreased compared to mESC injected group. Each value depicts mean ± SEM of number of rotation. PBS group vs mES group (*P<0.005), PBS group vs P-iPS group (*P<0.005).

To compare the therapeutic efficacy *in vivo* between mESCs and P-iPSCs in 6-week-old parkinsonian rats, we injected 400,000 neural precursor cells (stage 3) derived from either mESCs or P-iPSCs, or same volume of PBS into brain as control. The short-term behaviors of these animals were monitored by assessing apomorphine-induced turning behavior after 30 days post treatment (Figure S5A). Images were captured to make sure that cells remained in brain before sample harvest using Maestro1 (Figure S5B). The rat brain without any cell injection was compared with cell-injected brain to exclude the possibility of auto-fluorescence signals. As shown in Figure S5B, many cells remained around the injected region. Post-transplantation behavior data of animals showed that apomorphine-induced clockwise rotations were significantly reduced and improved in cell-injected animals compared to those injected with PBS, indicating both types of cell showed pronounced therapeutic effect. Interestingly, the average rotation score of P-iPSC group was slightly better than mESC group (190.7.±41.1 vs 219.7±24.9) (Figure 6E & Movie S1). To outrace the injected cells and differentiation status of grafted cells, we examined brain tissues by performing immunofluorescence staining. It revealed that majority of cells tagged with NEO-STEM^TM^ co-expressed TH/Tuj1, TH/VMAT2, or TH/Pitx3 indicating that injected cells were able to differentiate into neurons in vivo as well (Figure 6A,B, and C). Transplanted mESCs-derived neural precursor cells were assembled together in smaller numbers in brain, whereas transplanted P-iPSCs-derived precursors were abundant and dispersed widely from injected site *in vivo.* For quantification of the engrafting cells with neuro-specific markers in brain, we counted number of Tuj1-, Nurr1-, VMAT2-or TH-positive cells divided by number of whole DAPI-positive cells in the three random fields (Figure 6D). The average ratio of cells positive for specific mDA marker/cells positive for DAPI in mESC group was 20±3% while the score in P-iPSC group was 27±3%, indicating that the engraftment efficiency or survival in rat brain was better in neural precursor cells from P-iPSCs than from mESCs. The greatest difference between two groups was observed in the ratio of the cells positive for VMAT2 among total DAPI positive cells (24±5% vs. 35±7%), whereas the small difference was observed in ratio of the cells positive for other markers between mESC and P-iPSC groups. The ratio of cells positive for TH among total cells was 22±3% vs. 28±3% in mESCs and P-iPSCs, respectively. Such a higher number of the engrafted cells after injection *in vivo* in P-iPSC group may be attributable to the greater anti-apoptotic potential in addition to the better differentiation ability. Therefore, we investigated whether gene expression levels of Bcl-2 families, which act as an anti-apoptotic factor during CNS development [33], might be higher and allow a larger number of DA neurons to survive in P-iPSC groups than in mESCs during differentiation. In order to prove this hypothesis, we performed RT-PCR to evaluate the expression levels of anti-apoptotic Bcl-2 family genes. In the undifferentiated status, Bcl-xL expression was substantially higher in P-iPSCs than mESCs, whereas expression of Bcl-2 was comparable to each other. Intriguingly, upon differentiation into neural precursor cells, the expression of these two genes was greatly increased in both P-iPSCs and mESCs, which was more prominent in P-iPSCs than in mESCs (Figure S4). We also monitored the effect of cell therapy on parkinsonian rats until 8 weeks and observed the therapeutic effects similar to those at 2 weeks (Figure S5C). We did not find any tumor in either group of rats until 8 weeks after cell injection (PBS n = 3, mESC, P-iPSC groups n = 5). Taken together, these results from animal experiments are consistent with *in vitro* data, demonstrating that both mESCs and P-iPSCs had therapeutic potential for parkinsonian rat model with greater effect in P-iPSCs than in mESCs.

## Discussion

Stem cell has become one of the enchanting agents to cure neurodegenerative diseases. However, there are several issues that we have to overcome before applying stem cells in clinical field as a therapeutic modality, including genetic stability, tumorigenicity and immune rejection after transplantation. Although the emergence of iPSCs sheds light on overcoming the limitations of ESCs in cell therapy, it is necessary to approach carefully before clinical application. Many recent reports gave a warning that iPSCs derived by the use of retroviruses or lentiviruses have chromosomal integration of exogenes leading to unpredictable genetic dysfunction and expression of residual exogenes [34]. Recently, we succeeded in the generation of P-iPSCs, protein-based iPSCs, by treating mouse somatic cells with cell lysates of mESCs without genetic manipulation [9]. The 30 chimeric mice made of P-iPSCs have not developed any tumor during more than 1 year of breeding, suggesting the safety or genetic stability of protein-based iPSCs.

**Figure 7:**
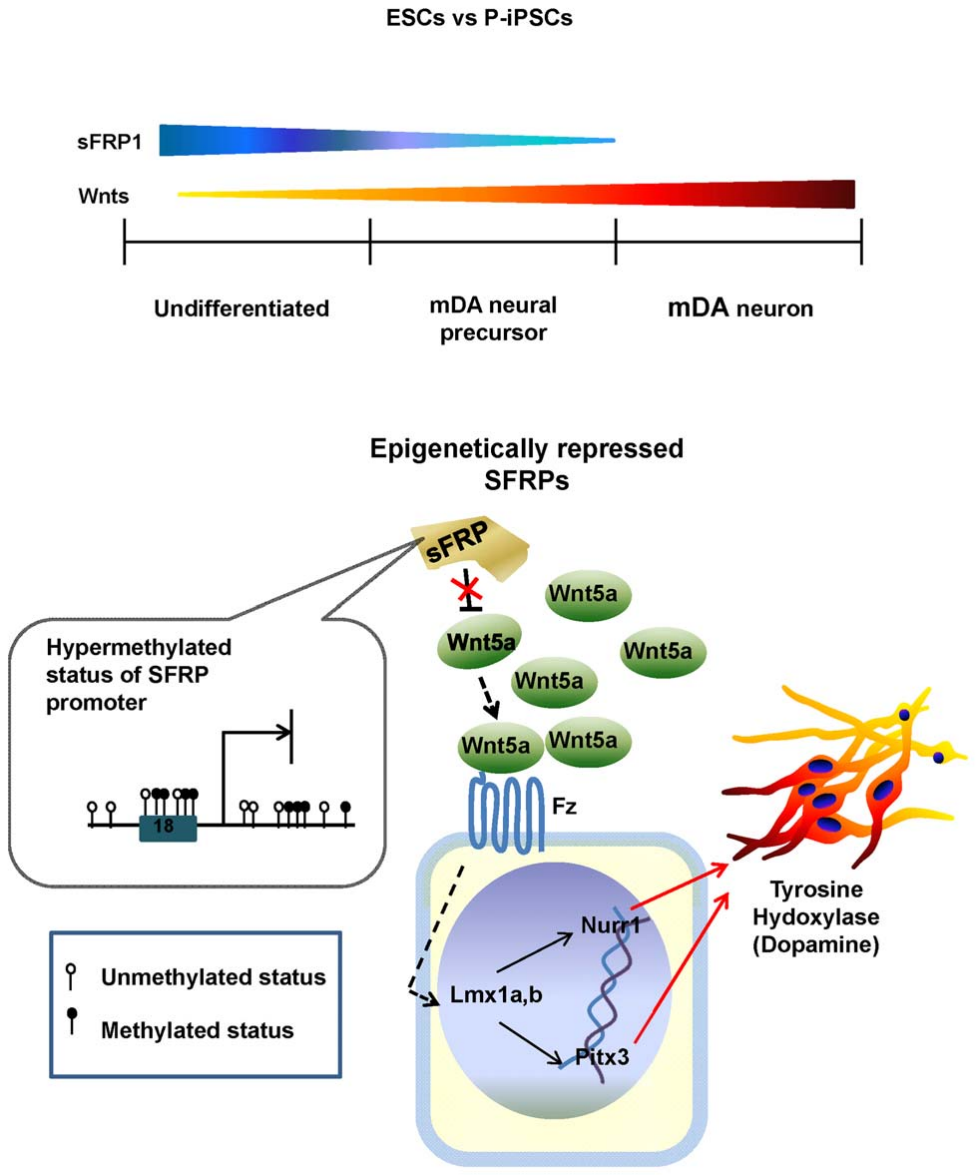
Schematic figure of comparative mechanism of mDA neuron differentiation between mESC and P-iPSC. The SFRP1 level is the highest in undifferentiated cells. As differentiation begins, SFRP1 expression was decreased by specific methylation at 17^th^, 18^th^, 21^st^, and 22^nd^ CpGs of its promoter. Epigenetically repressed SFRP1 gene fails to antagonize Wnt5a, which enables to augment Wnt5a signal transduction. Increased level of Wnt5a binds to the Frizzled (Fz) receptor and propagates dopamine neuron differentiating-signals by stimulating Lmx1a, b, Nurr1, Pitx3 and finally inducing tyrosine hydroxylase enzyme to specify neural precursor cells to mDA neurons.

Here, we investigated whether P-iPSCs could differentiate into DA neurons as observed in mESCs, has higher safety compared to genetically modified iPSCs as expected, and could serve as a valuable cell source for treatment of Parkinson's disease. Since there have been no studies to compare the differentiation efficiency between ESCs and P-iPSCs, we examined whether our P-iPSCs had similar characteristics to become DA neurons as ESCs both *in vitro* and *in vivo*. Our present results demonstrate that P-iPSCs had a higher differentiation potential to become DA neurons than mESCs. It had been extensively studied which factors are important in DA neural development during embryogenesis [35], [36]. We compared expression patterns of genes related with dopamine neuron during neuronal differentiation of P-iPSCs and mESCs. First, we found that the expression levels of overall genes at mRNA and protein levels of molecules associated with DA differentiation were higher in P-iPSCs than mESCs. Second, when we compared global gene expression and genome-wide histone modification between mESCs and P-iPSCs, the expression levels of Wnt family genes; *Wnt1*, *Wnt3a* and *Wnt5a*, were found to be higher in P-iPSCs than in mESCs. Wnt protein is known to control development of the nervous system by canonical and non-canonical pathways and it regulates axonal remodeling [37], path finding [38], and neuronal connectivity [24]. Elevated levels of all three Wnt genes would greatly endow P-iPSCs with a higher capacity to differentiate into DA neurons [29], [39]. It is noteworthy in our data that the Wnt5a was highly expressed starting from neural precursor cells of P-iPSCs to matured DA neurons and its expression level was higher in P-iPSCs than in mESCs. It has been reported that Wnt5a strongly influences initial neurite elongation and DA neuron survival. It also act as an inducer to transform Nurr1-positive NPCs to DA neurons through three different pathways; Wnt/ß-catenin canonical, Wnt/calcium/non-canonical, and Wnt/planar cell polarity (PCP)/non-canonical pathways [40], [41]. Considering these previous reports and our results together, we could deduce that Wnt5a is the major factor that intrinsically potentiates P-iPSCs to differentiate into DA neurons more than mESCs. The summary of our findings is illustrated in the schematic figure (Figure 7).

Since hypermethylation of promoter down-regulates its gene expression, we compared methylation patterns of SFRP promoter between ESCs and P-iPSCs. Although methylation on SFRP promoter was higher in P-iPSCs than mESCs, it was not enough to explain the significant differences in gene expression levels observed between two cell types. Interestingly, we found that 17^th^, 18^th^, 21^st^, and 22^nd^ CpG sites were specifically hypermethylated in P-iPSCs, whereas not observed in mESCs. We screened transcription factors which bind to these CpG sites by using of TRNASFAC (BIOBASE) data base. ETF (Epidermal growth factor receptor specific transcription factor) and PBX (Pre-B-cell leukemia homeobox) could bind to 17^th^, 18^th^, 21^st^, and 22^nd^ CpG sites, and might regulate SFRP gene expression (data not shown). ETF usually binds TATA box-lacking and CG-rich promoter such as EGFR (Epidermal growth factor receptor) [42]. Since promoter of SFRP also showed typical TATA box-lacking and CG-rich promoter, ETF might be a strong candidate for regulation of SFRP promoter. There are three isotypes of PBX, including PBX1, 2 and 3. PBX2 and PBX3 are 92% and 94% homologous to PBX1 but all three proteins diverged significantly in their amino- and carboxy- termini sequences [43]. According to our microarray data, PBX 1 and 3 were highly expressed in somatic cells, whereas PBX2 was expressed in two times higher in mESCs and P-iPSCs than in somatic cells (data not shown). Therefore, PBX2 could be a good candidate to regulate SFRP promoter as well. Based on these findings, methylation of specific CpG sites on SFRP promoter might decrease the expression of SFRP and increases Wnt5a signal transduction activity. However, our studies only provided a glimpse of explaining higher differentiation efficacy in P-iPSCs, suggesting that further investigation is needed to support our hypothesis concretely.

It is well-established that families of SFRP are important in DA neuron development [44], [45]. However, we majorly focused on expression pattern of SFRP1 in our study because it is known to interact with Wnt5a in the epithelium of developing gut [46]. Therefore, we investigated expression pattern of SFRP1 and found that SFRP1 was more predominantly down-regulated throughout mDA neuron differentiation in P-iPSCs compared to mESC, unveiling the mechanism of higher mDA differentiation capacity observed in P-iPSC.

Finally, we evaluated whether our own P-iPSCs could be a suitable source for treatment of Parkinson's disease through animal studies. Following transplantation into PD animal models, neural precursor cells derived from P-iPSCs significantly relieved symptoms in two weeks of follow-up, which maintained up to 8 weeks, compared with control animals injected with PBS. Such a higher efficacy was correlated with a larger number of differentiated cells in grafted site in P-iPSC than in mESC group. Moreover, we also confirmed the safety by demonstrating that transplantation of proper numbers (400,000 cells) of P-iPSC-derived NPCs did not result in tumor formation and reduced parkinsonian symptom until after eight weeks of follow-up (Figure S5C).

This new type of protein-based and highly Wnt5a activated iPSCs could serve as a promising cell source for DA neurons as efficient as, or even more efficient than mESCs. This strategy may provide the efficient and safe patient-specific therapy for PD without any ethical problems or safety issues.

## Supporting Information

Figure S1 **Nestin-positive cells in mESC and P-iPSC.**S3 cells of mESC and P-iPSC groups were observed after 7 days cultured in ITSFn media, most of cells were nestin-positive. And more Nestin-positive cells were observed in P-iPSCs. Scale bars  = 20 μm.
(TIFF)

Figure S2 **Microarray analysis and Genome-wide ChIP-Seq analysis with methylation status of H3K4 and H3K27 in mESCs and P-iPSCs.** (A) The gene expression and histone modification profiles were shown using 185 genes related with Wnt signaling pathway among neuron differentiation-related genes. Data was shown in Log2 fold change values (P-iPSC/mESC). (B) The gene expression and histone modification profiles for Wnt gene family. H3K4 trimethylation were enriched at Wnt5a promoter in P-iPSCs. Analysis of genome-wide histone modification between mESCs and P-iPSCs showed that there was no difference in the pattern of H3K4 trimethylation in both cell types while H3K27 modification was slightly higher in mESCs. (C) Analysis of genome-wide histone modification between mESCs and P-iPSCs showed that H3K4 trimethylation pattern was not different in both cells but H3K27 modification was slightly divergent, especially in upstream of transcription start site.
(TIFF)

Figure S3 **Co-localization and gene expression analysis of TH and Wnt5a-positive cells of mESCs and P-iPSCs.** Wnt5a was well co-localized with TH and analysis of changes in Wnt expression of mESCs and P-iPSCs revealed three types of Wnts emerged sequentially as in embryogenesis. (A) More TH-positive cells existed in P-iPSC groups and 100% overlaying TH/Wnt5a expression shows Wnt5a expression may lead neural precursor cells into TH-positive cells. (n =  3, ** P<0.01). (B) RT-PCR data shows changed levels of neurogenesis related-Wnts during differentiation into mDA neurons in mESCs and P-iPSCs. In contrast to an increase of Wnt signals, the Wnt antagonist SFRP1 expression was reduced at the same time. In P-iPSCs, the level of Wnts was higher whereas SFRP1 expression was lower compared to levels in mESCs.
(TIFF)

Figure S4 **Increased anti-apoptotic gene level leads to higher cell survival of P-iPSCs after cell transplantation.** mRNA level of anti-apoptotic genes significantly changed during cell differentiation. Bcl-2 and Bcl-xL were expressed in neural precursor cells of mESCs and P-iPSCs. The higher expression levels of these genes in P-iPSCs than in mESCs may support the result that the higher number of neuronal precursor cells derived from P-iPSCs than mESCs survived after transplantation to brain.
(TIFF)

Figure S5 **Time table for** ***in vivo*** **study.** (A) Time table of in vivo experiment of mDA neuronal differentiation for eight-weeks. (B) Before harvest of brain from rats, cell existence was identified by MAESTRO I imaging system. In comparison to vehicle-injected brain, signals were detected only in the brain injected with the labeled cells. (C) The efficiency of cell therapy was monitored on parkinsonian rats until 8 weeks, which demonstrated that neural precursor cells derived from mESCs (n = 5) or P-iPSCs (n = 5) had therapeutic potential. (PBS n = 3).
(TIFF)

Movie S1 **Post-transplantation behavior data of animals showed that apomorphine-induced clockwise rotations were significantly reduced and improved in cell-injected animals compared to those injected with PBS, indicating both types of cell showed pronounced therapeutic effect.**
(RAR)

Table S1 **List of primers to detect target genes.**
(TIFF)
