# Supplementary figures and images for: Comparative Study of Efficacy of Dopaminergic Neuron Differentiation between Embryonic Stem Cell and Protein-Based Induced Pluripotent Stem Cell
Source: PLoS One. 2014 Jan 22;9(1):e85736. doi: 10.1371/journal.pone.0085736 (PMC3899054; doi:10.1371/journal.pone.0085736)

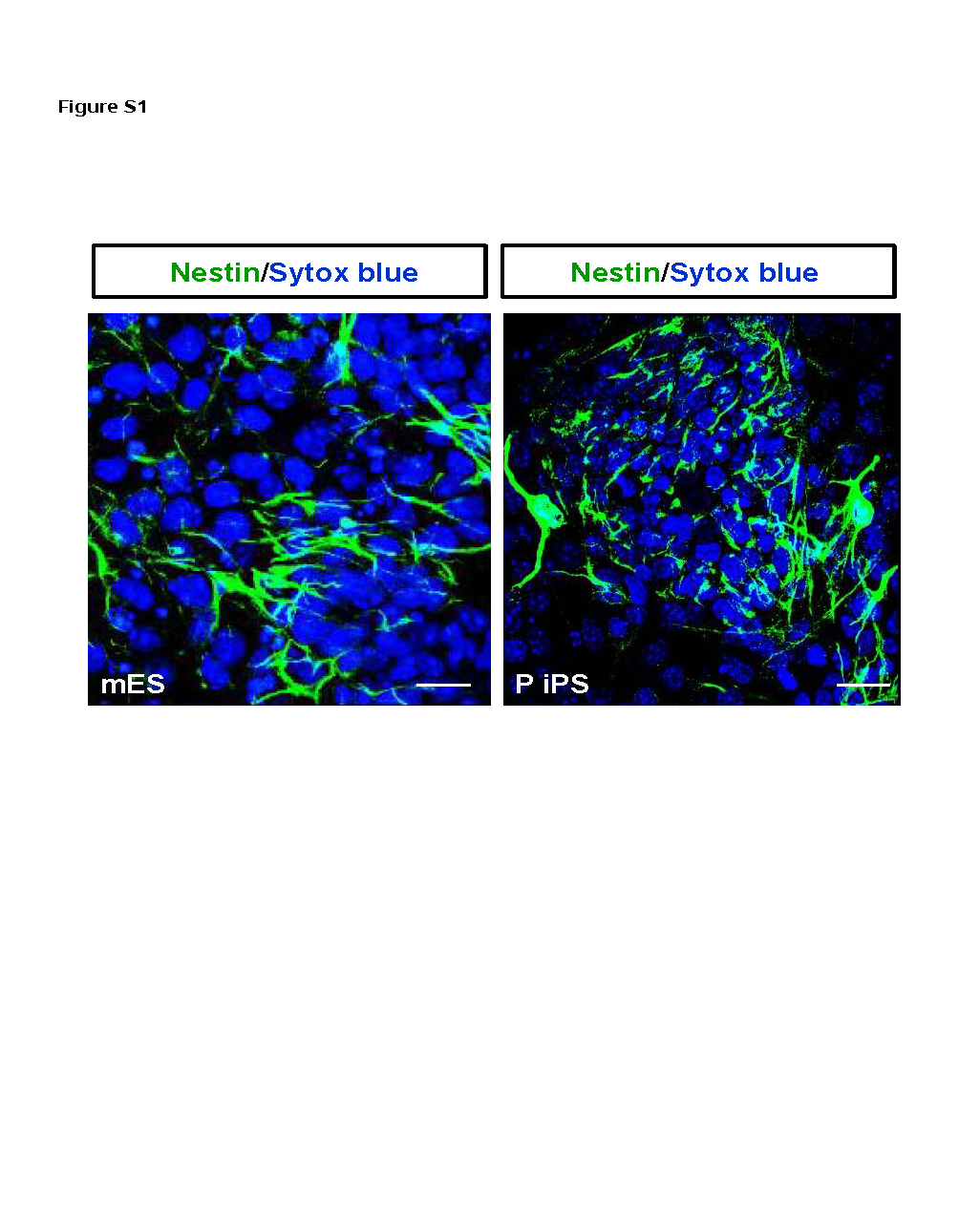

Supplement: Figure S1 — Nestin-positive cells in mESC and P-iPSC.S3 cells of mESC and P-iPSC groups were observed after 7 days cultured in ITSFn media, most of cells were nestin-positive. And more Nestin-positive cells were observed in P-iPSCs. Scale bars = 20 μm. (TIFF) [file pone.0085736.s001.tiff]

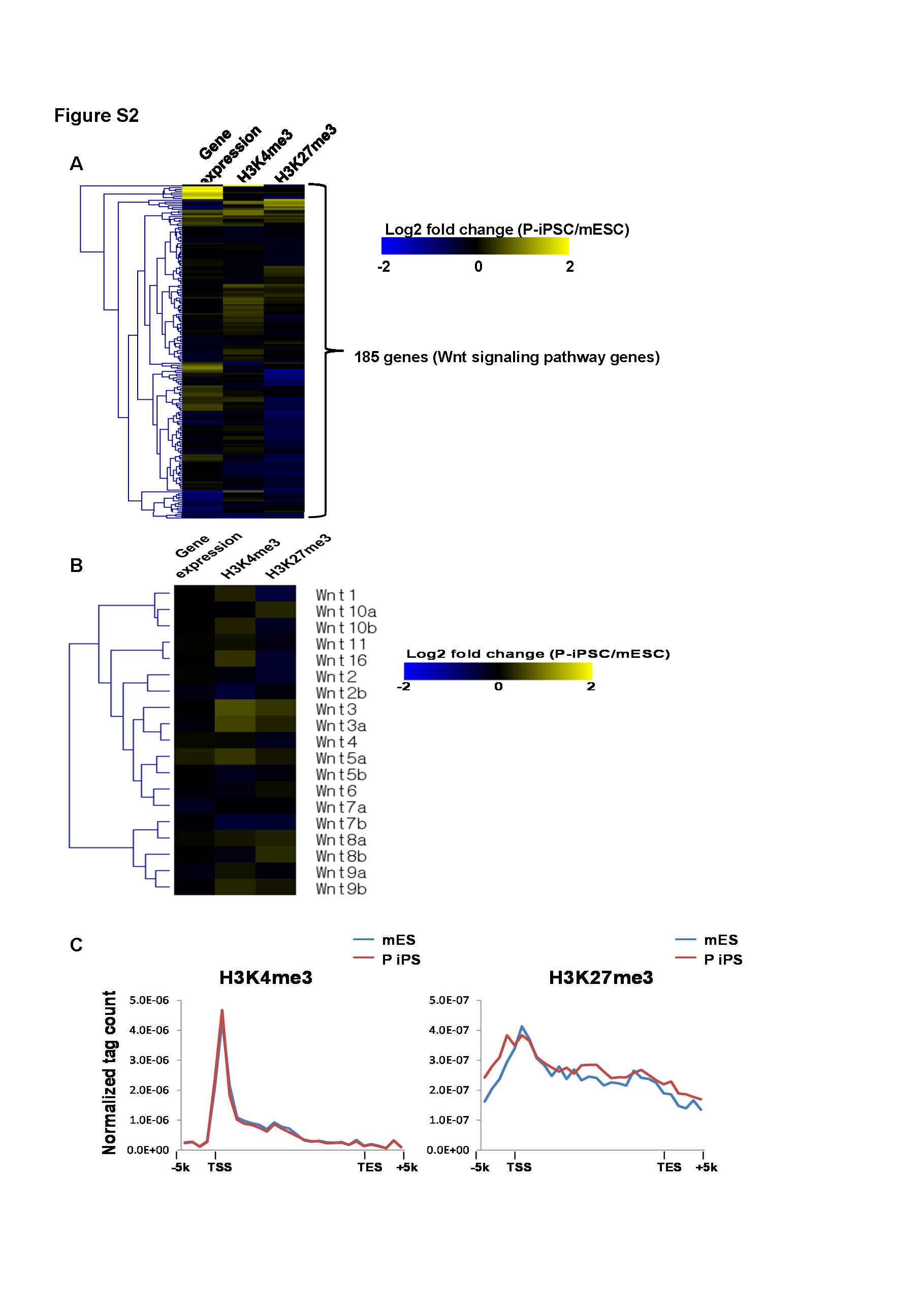

Supplement: Figure S2 — Microarray analysis and Genome-wide ChIP-Seq analysis with methylation status of H3K4 and H3K27 in mESCs and P-iPSCs. (A) The gene expression and histone modification profiles were shown using 185 genes related with Wnt signaling pathway among neuron differentiation-related genes. Data was shown in Log2 fold change values (P-iPSC/mESC). (B) The gene expression and histone modification profiles for Wnt gene family. H3K4 trimethylation were enriched at Wnt5a promoter in P-iPSCs. Analysis of genome-wide histone modification between mESCs and P-iPSCs showed that there was no difference in the pattern of H3K4 trimethylation in both cell types while H3K27 modification was slightly higher in mESCs. (C) Analysis of genome-wide histone modification between mESCs and P-iPSCs showed that H3K4 trimethylation pattern was not different in both cells but H3K27 modification was slightly divergent, especially in upstream of transcription start site. (TIFF) [file pone.0085736.s002.tiff]

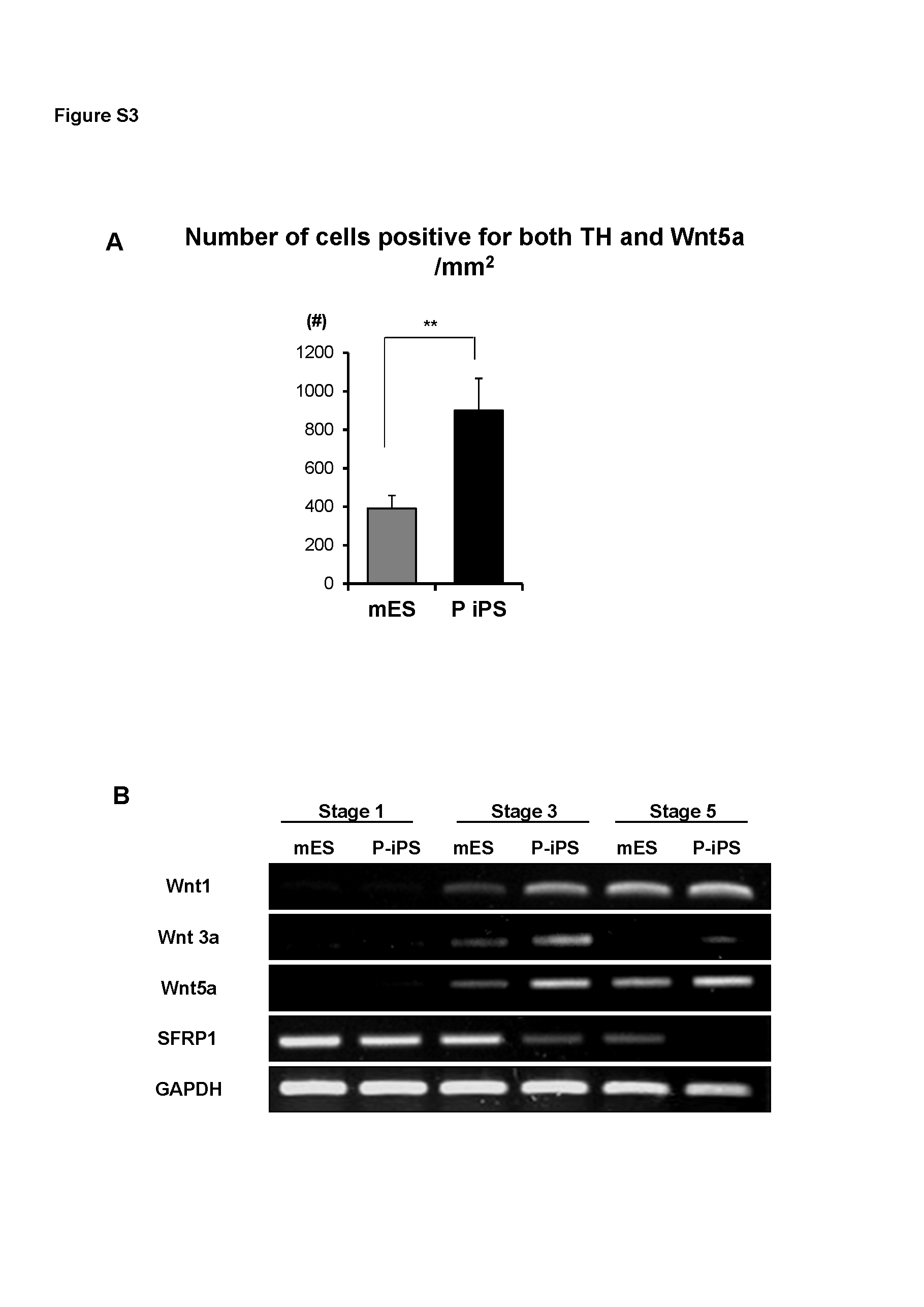

Supplement: Figure S3 — Co-localization and gene expression analysis of TH and Wnt5a-positive cells of mESCs and P-iPSCs. Wnt5a was well co-localized with TH and analysis of changes in Wnt expression of mESCs and P-iPSCs revealed three types of Wnts emerged sequentially as in embryogenesis. (A) More TH-positive cells existed in P-iPSC groups and 100% overlaying TH/Wnt5a expression shows Wnt5a expression may lead neural precursor cells into TH-positive cells. (n = 3, ** P<0.01). (B) RT-PCR data shows changed levels of neurogenesis related-Wnts during differentiation into mDA neurons in mESCs and P-iPSCs. In contrast to an increase of Wnt signals, the Wnt antagonist SFRP1 expression was reduced at the same time. In P-iPSCs, the level of Wnts was higher whereas SFRP1 expression was lower compared to levels in mESCs. (TIFF) [file pone.0085736.s003.tiff]

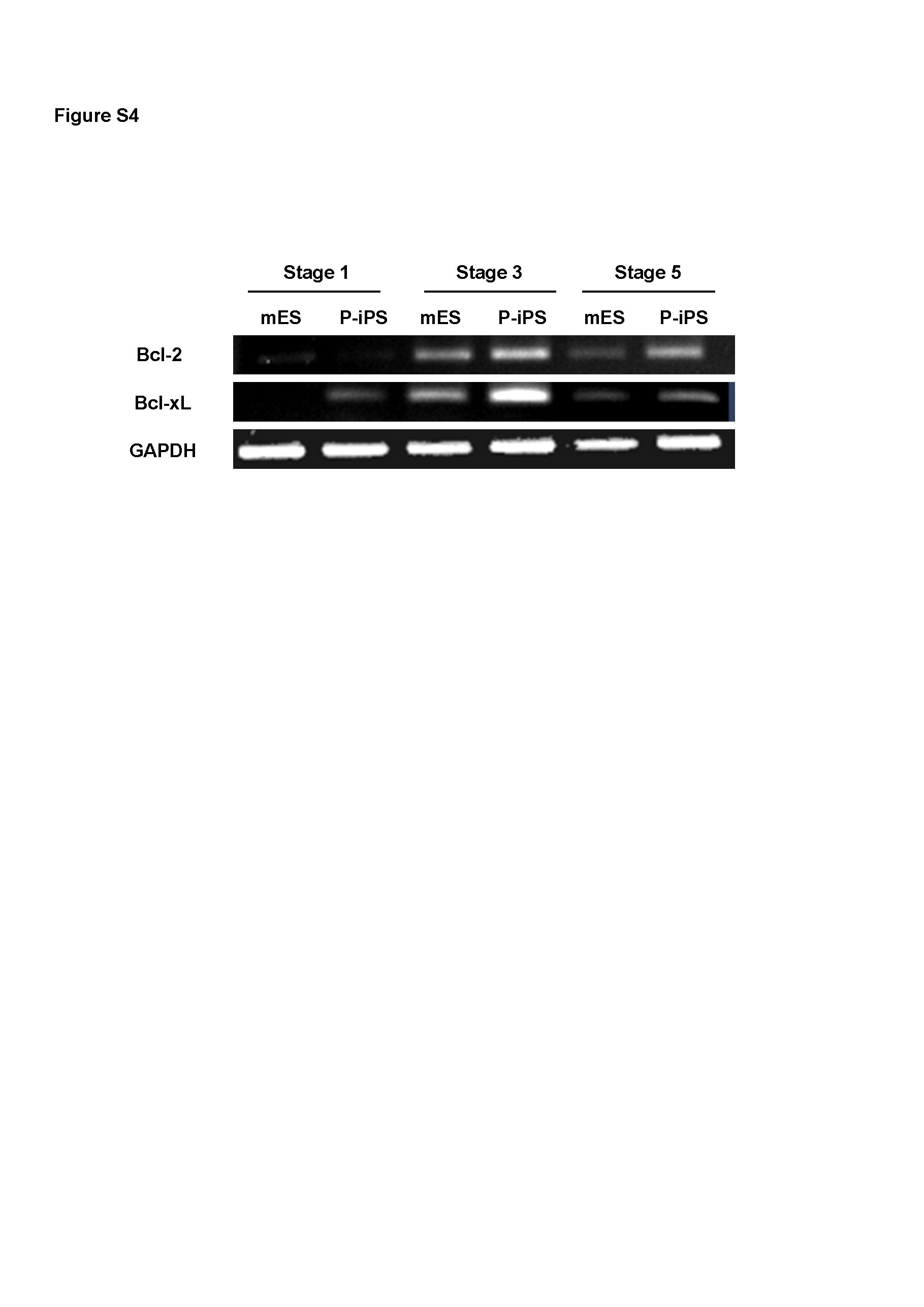

Supplement: Figure S4 — Increased anti-apoptotic gene level leads to higher cell survival of P-iPSCs after cell transplantation. mRNA level of anti-apoptotic genes significantly changed during cell differentiation. Bcl-2 and Bcl-xL were expressed in neural precursor cells of mESCs and P-iPSCs. The higher expression levels of these genes in P-iPSCs than in mESCs may support the result that the higher number of neuronal precursor cells derived from P-iPSCs than mESCs survived after transplantation to brain. (TIFF) [file pone.0085736.s004.tiff]

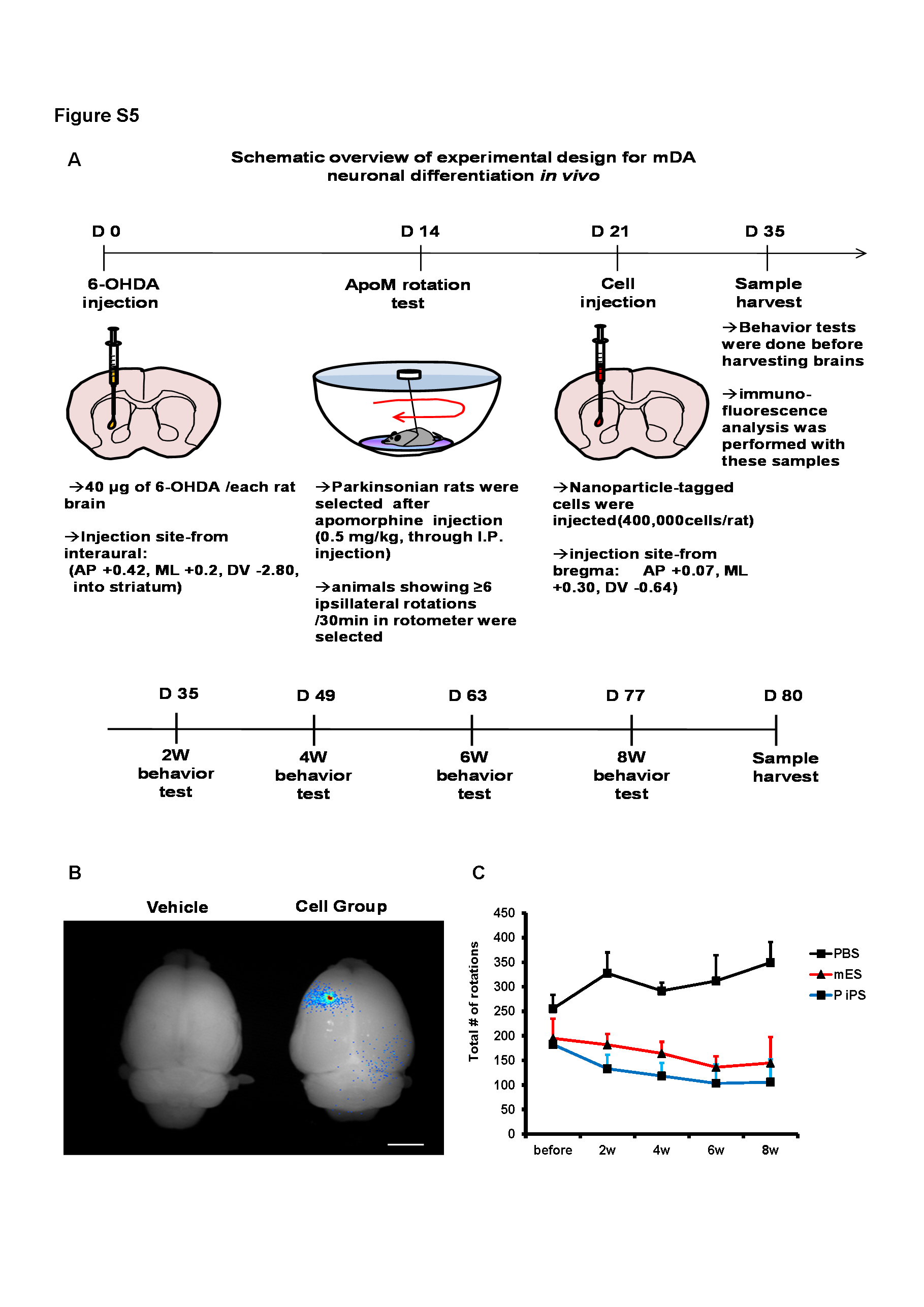

Supplement: Figure S5 — Time table for in vivo study. (A) Time table of in vivo experiment of mDA neuronal differentiation for eight-weeks. (B) Before harvest of brain from rats, cell existence was identified by MAESTRO I imaging system. In comparison to vehicle-injected brain, signals were detected only in the brain injected with the labeled cells. (C) The efficiency of cell therapy was monitored on parkinsonian rats until 8 weeks, which demonstrated that neural precursor cells derived from mESCs (n = 5) or P-iPSCs (n = 5) had therapeutic potential. (PBS n = 3). (TIFF) [file pone.0085736.s005.tiff]
